# 3D Printing On-Water Sports Boards with Bio-Inspired Core Designs

**DOI:** 10.3390/polym12010250

**Published:** 2020-01-20

**Authors:** Aref Soltani, Reza Noroozi, Mahdi Bodaghi, Ali Zolfagharian, Reza Hedayati

**Affiliations:** 1 Department of Engineering, School of Science and Technology, Nottingham Trent University, Nottingham NG11 8NS, UK; aref.soltani@me.iut.ac.ir (A.S.); reza.noroozi@ut.ac.ir (R.N.); 2 Department of Mechanical Engineering, Isfahan University of Technology, Isfahan 8415683111, Iran; 3 School of Mechanical Engineering, Faculty of Engineering, University of Tehran, Tehran 1417466191, Iran; 4 School of Engineering, Deakin University, Geelong, VIC 3216, Australia; a.zolfagharian@deakin.edu.au; 5 Novel Aerospace Materials Group, Faculty of Aerospace Engineering, Delft University of Technology (TU Delft), Kluyverweg 1, 2629 HS Delft, The Netherlands; r.hedayati@tudelft.nl

**Keywords:** 3D printing, bio-inspired design, sports engineering, surfboard, kiteboard, FEM

## Abstract

Modeling and analyzing the sports equipment for injury prevention, reduction in cost, and performance enhancement have gained considerable attention in the sports engineering community. In this regard, the structure study of on-water sports board (surfboard, kiteboard, and skimboard) is vital due to its close relation with environmental and human health as well as performance and safety of the board. The aim of this paper is to advance the on-water sports board through various bio-inspired core structure designs such as honeycomb, spiderweb, pinecone, and carbon atom configuration fabricated by three-dimensional (3D) printing technology. Fused deposition modeling was employed to fabricate complex structures from polylactic acid (PLA) materials. A 3D-printed sample board with a uniform honeycomb structure was designed, 3D printed, and tested under three-point bending conditions. A geometrically linear analytical method was developed for the honeycomb core structure using the energy method and considering the equivalent section for honeycombs. A geometrically non-linear finite element method based on the ABAQUS software was also employed to simulate the boards with various core designs. Experiments were conducted to verify the analytical and numerical results. After validation, various patterns were simulated, and it was found that bio-inspired functionally graded honeycomb structure had the best bending performance. Due to the absence of similar designs and results in the literature, this paper is expected to advance the state of the art of on-water sports boards and provide designers with structures that could enhance the performance of sports equipment.

## 1. Introduction

Mechanical design and modeling are the most recent methods for tackling technical concerns in the field of sports science and could have various benefits such as injury prevention, reduction of the cost of manufacturing, minimization of the weight, and enhancement of the performance of the equipment. For example, Caravaggi et al. [1] developed and tested a novel cervical spine protection device to keep the athlete’s neck in its safe physiological range. Shimoyama et al. [2] employed a finite element method (FEM) to optimize the design of the sports shoe sole, followed by lightening the sole weight. Furthermore, Sakellariou et al. [3] applied coupled algorithms with the FLUENT^®^ solver to optimize a surfboard fin shape, resulting in maximum lift per drag ratio. With regard to head protection, Mosleh et al. [4] employed an FEM to investigate the structural parameters of a bicycle helmet, aiming to improve the performance of composite foam in reducing rotational velocity and acceleration during indirect impact. Gudimetla et al. [5] exploited computational fluid dynamics (CFD) to analyze both lift and drag coefficient for the three- and four-fin surfboards per angle of incidence at different velocities. It was shown that the highest lift and drag for the three-fin arrangement occurred at a smaller angle of incidence compared to the four-fin design.

Boarding on the water including surfboarding, kiteboarding, and skimboarding is an action sport in which the sportsman utilizes a special board to ride across the water. In all of these waterboarding sports, the board is the main instrument that keeps the rider on the water; however, some differences exist from one to another. For instance, in kiteboarding, the kite is the source of the motive force, whereas surfboarding uses the force of the moving surf. In the late 1970s, providing a substantial vertical component of force, sailboarders replaced their conventional sails with modern kites. This enables speeds to be over 100 km/h and jump with more than 50 m in height and 250 m in distance [6]. As a result of these destructive interactions and consequent forces, many kiteboards have been broken. Likewise, large force impulses generated by impacting waves often result in surfboard fractures. The pieces of broken boards are harmful to both the environment and humans, especially when toxic materials like fiberglass are used in the production process and considering that more than 400,000 boards are sold every year worldwide [7]. Consequently, researchers have turned their attention to bio-degradable materials and highly stiff structures, which subsequently demand more advance manufacturing by conventional manufacturing methods [8].

Modern boards are mostly manufactured with a foam or a lightweight core covered by the top and bottom shells, called a sandwich structure [9]. This lightweight core provides boards with not only a high resistance to bending, preventing boards from breaking, but also an increase in buoyancy, stability, and improved user experience [6,10]. Recently, various cellular core structures have been introduced for different purposes. Platek et al. [11] tested gradient three-dimensional (3D)-printed structures to compare their tensile properties. Compton et al. [12] fabricated various composite structures and tested them under compression. Among all of these structures, a bio-inspired honeycomb structure, due to its high stiffness per weight ratio, has received much attention [10,13,14,15]. However, structural complexity has rendered these composites challenging to manufacture.

3D printing, or additive manufacturing (AM), refers to producing objects from a 3D model, mostly layer by layer. The main benefits of 3D printing could be listed as design freedom, waste diminution as well as the ability to fabricate complex structures with sufficient geometrical precision [12,13,16,17,18,19,20]. Recently, studies have been conducted to take advantage of 3D printing in the field of sports. For instance, Gately et al. [21] employed 3D printing and CFD to fabricate a commercially usable surfboard fin. Using a fused deposition modeling (FDM) type 3D printer, Park et al. [22] fabricated an optimized rifle support for the sport biathlon. The initial design showed a 12.7% stiffness improvement and 23%–43% structural safety enhancement in the printing direction. The final design, however, was manufactured using FDM, and the results showed a 40% improvement in critical force under the compression test. Cazón-Martín et al. [23] designed and additively manufactured a lattice structure shin pad for football players by using a multi-material 3D printing apparatus. Novel shin pads demonstrated a reduction in impact acceleration between 42% and 68% when compared to traditional shin pads. In addition, with regard to penetration, additively manufactured shin pads were improved from 13% to 32%, while the attenuation and contact times were identical. 

The literature shows that in previous studies, the researchers mostly studied the board geometry and that they only made slight modifications to the traditional boards. In this paper, inspired by nature, different patterns were introduced for the internal core structure of the board. First, FDM, as the most widely used 3D printing technology, was employed to fabricate a board with a uniform honeycomb core from polylactic acid (PLA), then an experimental three-point bending test was carried out to investigate its bending performance. Afterward, a geometrically linear analytical solution and non-linear FEM software package ABAQUS were implemented for modeling the deformation of the uniform honeycomb structure under a three-point bending test. After the validation of the numerical tool with the experimental results, the accurate FEM tool was employed to simulate the board with different nature-inspired core structures such as a pinecone-inspired pattern, spiderweb-inspired pattern, carbon crystal lattices, and gradient honeycomb, all tested by the three-point bending test, while the total volume of the board was kept constant.

## 2. Materials and Methods 

### 2.1. Board Design (Uniform Honeycomb Sandwich Structure)

Modern boards are primarily composed of an inner foam core covered by a thin outer shell, generally called a sandwich structure [6,9]. The foam core enables reduced weight, increased buoyancy, and better stability for the rider, whilst the sandwich structure provides improved bending resistance. Such structures are mainly composed of three main parts: a top shell, a lightweight core, and a bottom shell [24]. The board in this study, however, was comprised of two primary parts, a top shell, and a merged bottom shell and lightweight core, which can be designed and 3D-printed with different patterns. Furthermore, most boards are manufactured with a bottom curvature that aims at better edging and upwind ability, which is significant for beginners, providing more grip and stability when compared to flat boards [25].

**Figure 1:**
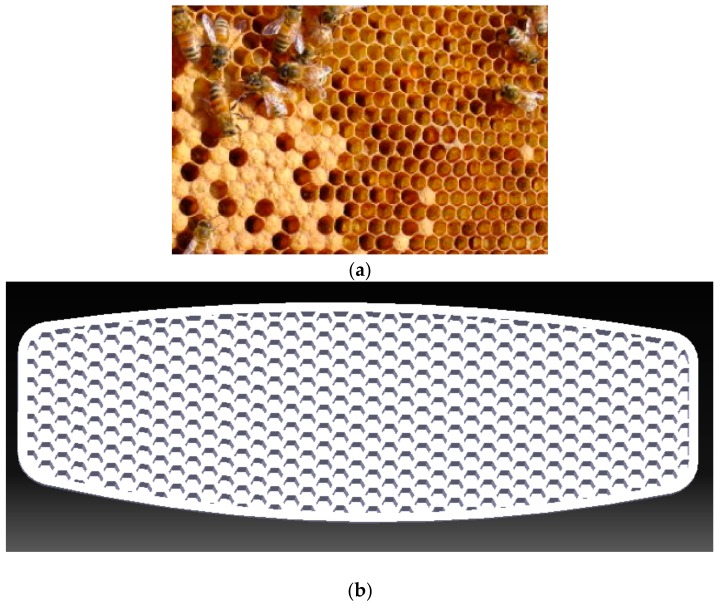

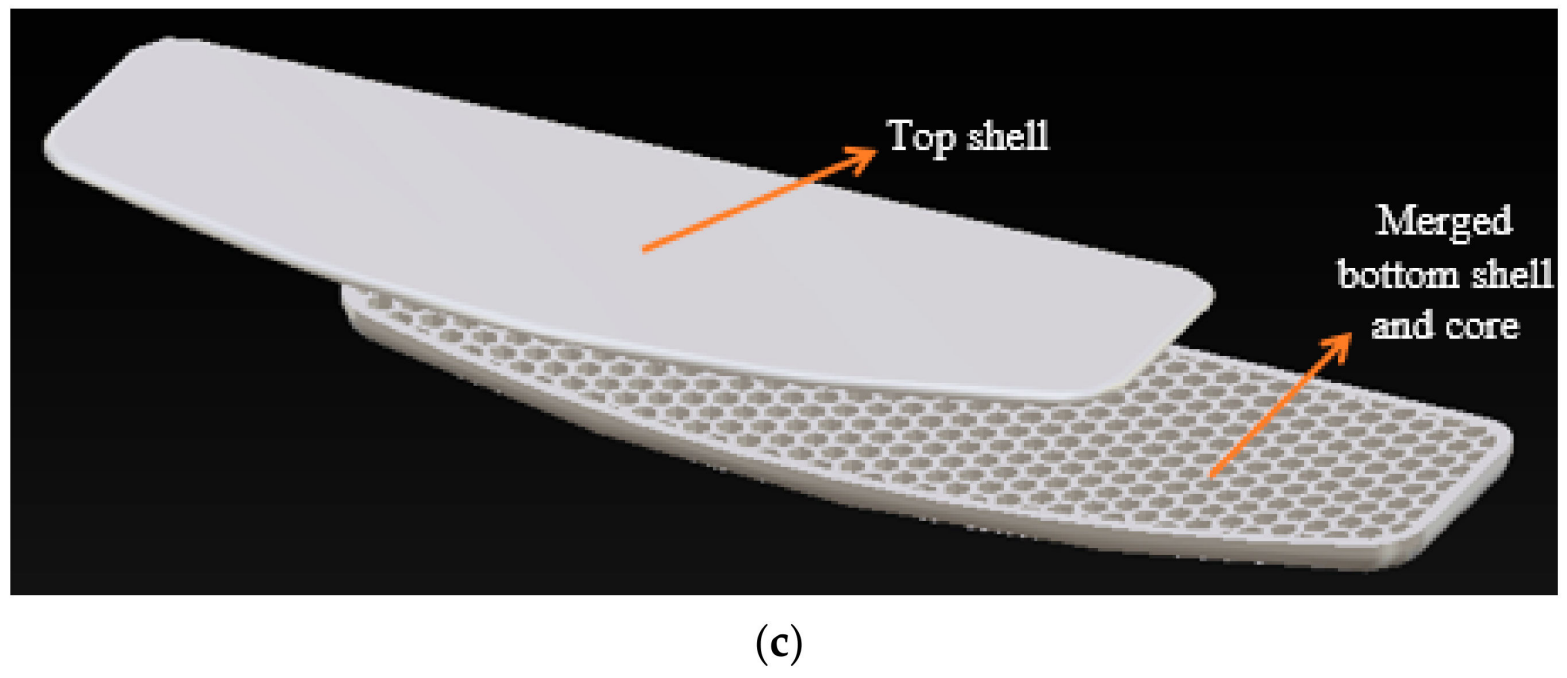
(**a**) A natural honeycomb structure; (**b**) the designed honeycomb core inspired by nature. (**c**) board components: the top shell, and the merged bottom shell and core.

In this study, inspiring by nature a primary structure for the board core was designed. As illustrated in Figure 1a, a beehive is made up of a regular pattern of hexagonal honeycomb cell structure, which was used to design the core of the board by implementing CATIA V5 software [26]. The designed honeycomb core structure and exploded 3D model are shown in Figure 1b,c, respectively.

**Figure 2:**
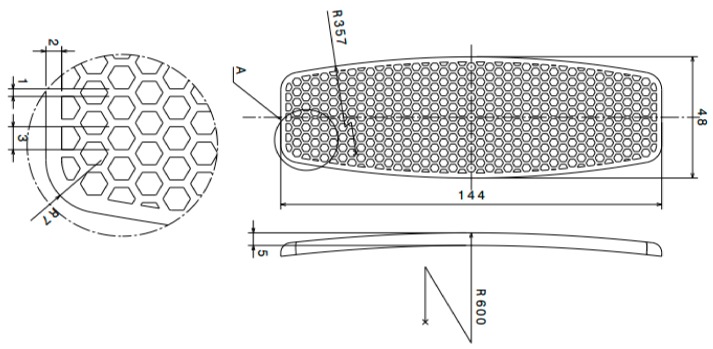
Geometrical dimensions of the board with a honeycomb structure core (all the dimensions are in mm).

A smaller scale version of a real on-water sports board was designed. The dimensions of the designed merged bottom shell and the honeycomb core board are presented in Figure 2. The board had a 48 mm width and 144 mm length with a 357 mm radius curvature at two sides. A bottom curvature of 600 mm was considered, resulting in a model closer to the real one. The hexagonal honeycomb structure formed the core of the board, and was repeated across the specimen. As can be seen in the detailed view, the 3 mm wide honeycombs were patterned with 1 mm thick walls. Moreover, the bottom and top shells of the board had thicknesses of 5 and 1.5 mm, respectively.

### 2.2. Materials and 3D Printing

**Figure 3:**
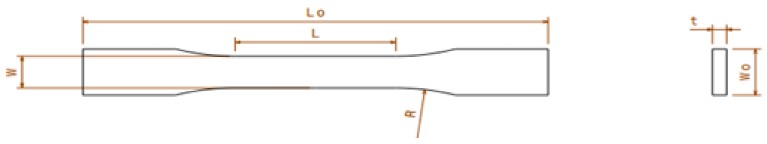
Sketch of TYPE 1 ASTM D638 specimen for the tensile test.

This section aims to experimentally determine the mechanical property of PLA fabricated by an FDM 3D printing apparatus. The guidelines of ASTM D638 [27], Standard Test Method for Tensile Properties of Plastics, were followed. Five different specimens are introduced in the standard, each having the same geometry, but with different dimensions as a function of thickness. The TYPE 1 specimen was chosen to design the tensile test dog-bone specimens. The sketch of the specimen and the dimensions are given in Figure 3 and Table 1, respectively.

The designed specimen with the square cross-section was fabricated using the XYZ da Vinci 1.0 Pro 3D printer, which works on the basis of FDM technology. In this technique, raw cylindrical thermoplastic filament is mechanically dragged into the melting nozzle, and the molten polymer is extruded on a heated platform known as the bed. After the first layer of the object is deposited, the nozzle moves upward to extrude the second layer on the previously printed layer; this process continues until the object is completely printed. The layer height is defined as the distance between any two sequential layers. The layer height is one of the most important FDM printing parameters with respect to the mechanical properties of the printed object [28]. For this study, PLA was used as the raw material of 3D printing. Consequently, the nozzle temperature of 230 °C, bed temperature of 40 °C, layer thickness of 0.2 mm, internal fill density of 100%, and printing speed of 20 mm/s were set. All the layers were combined with the raster angle of +45° and −45°.

**Figure 4:**
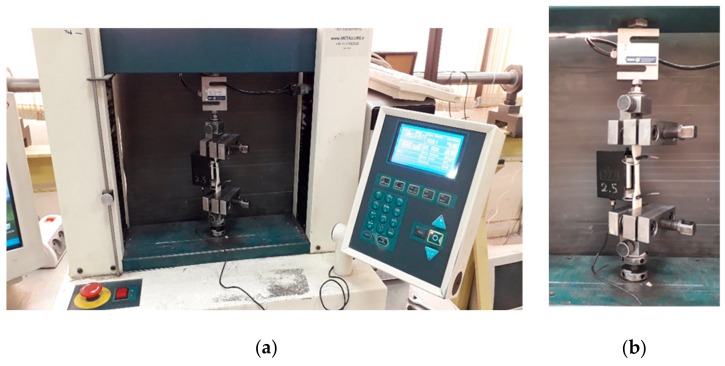
(**a**) Hounsfield-H25KS testing machine; (**b**) a dog-bone specimen under tensile test.

**Figure 5:**
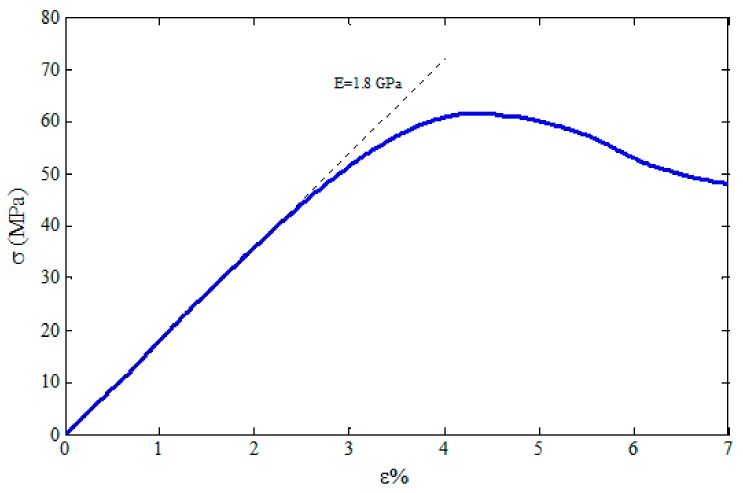
The stress–strain curve of the PLA tensile specimen.

In order to determine the Young’s modulus of the FDM 3D-printed PLA materials, a uniaxial tensile test was conducted using a Hounsfield-H25KS testing machine. The temperature was kept constant at 23 °C and the strain rate was set at 0.001/s to ensure that the test condition was quasi-static loading. The tensile mechanical bench machine and a specimen under test are shown in Figure 4. The stress–strain (σ-ε) curve for the tensile dog-bone sample is shown in Figure 5. The specimen exhibited a linear elastic behavior, followed by yielding and plastic deformation. The approximate values of the Young’s modulus and yield stress were determined to be 1.8 GPa (the slope of the linear region) and 60 MPa, respectively, followed by softening irrecoverable plastic deformation.

### 2.3. D Printing and Assembling of the Board with the Honeycomb Core Structure

**Figure 6:**
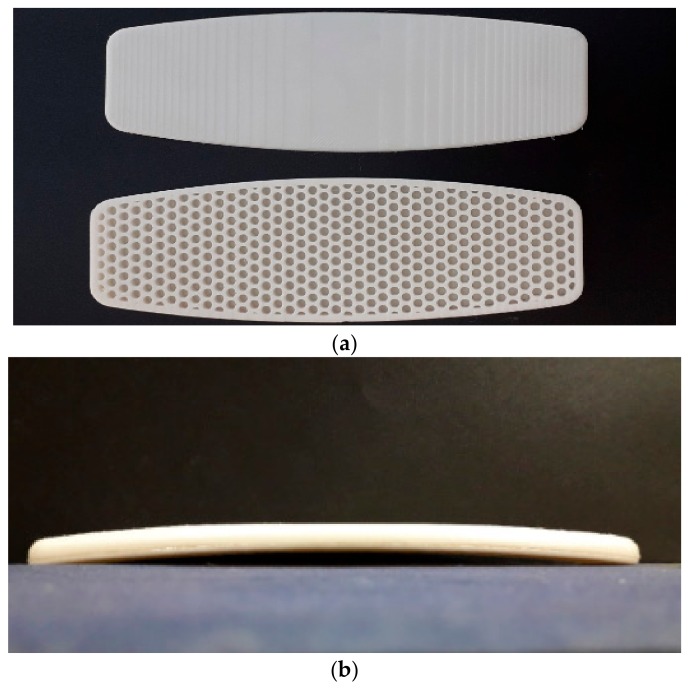
(**a**) Two separate 3D-printed parts of the board; (**b**) two parts glued together with strong adhesive.

In the next step, two parts of the board (bottom and top shell) were separately 3D printed in a smaller scale of real on-water sports board using PLA filament. The nozzle temperature, bed temperature, layer thickness, internal fill density, and printing speed were set to 230, 40 °C, 0.2 mm, 100%, and 20 mm/s, respectively. Both 3D-printed board parts are shown separately in Figure 6a, while Figure 6b depicts the two parts glued together with a strong adhesive after the 3D printing process.

### 2.4. Experimental Three-Point Bending Test of Uniform Honeycomb

**Figure 7:**
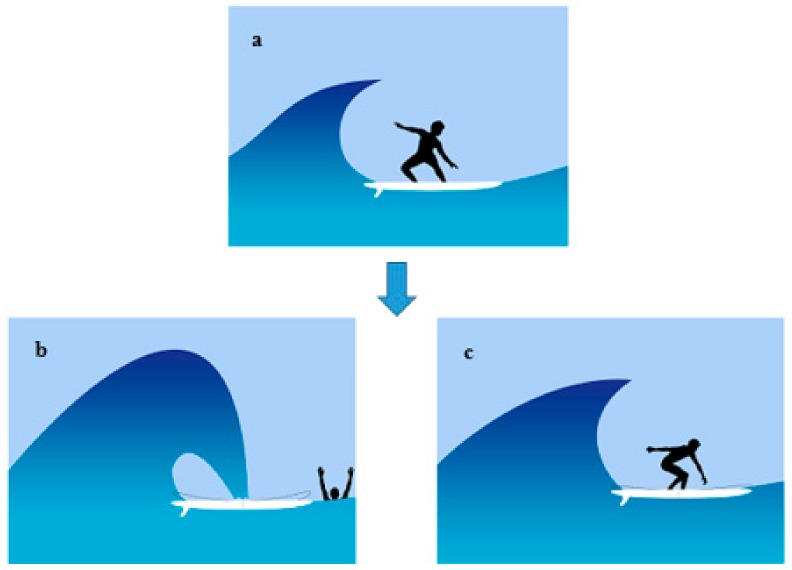
A schematic of the most common situations that boards break: (**a**) A standard boarding with a fine distance between the rider’s feet; (**b**) when the wave hits on the board while the rider falls in the water; (**c**) when the rider’s feet get so close together that the weight of their body is concentrated in the middle of the board.

One of the most common types of surfboard fractures takes place in the middle section of the board, between the feet of the surfer. These breaks occur in two main circumstances: the most frequent breakage takes place when the lip of the wave impacts in the middle the board, ripping it apart into two separate parts, just after the surfer falls in the water (Figure 7b); the second type of breakage occurs when the feet of the surfer get close together, concentrating the pressure of the body in the middle of the board (see Figure 7c) [29]. In both of these circumstances, an immense force acts upon the middle portion of the board, causing large bending stress that may result in breakage.

**Figure 8:**
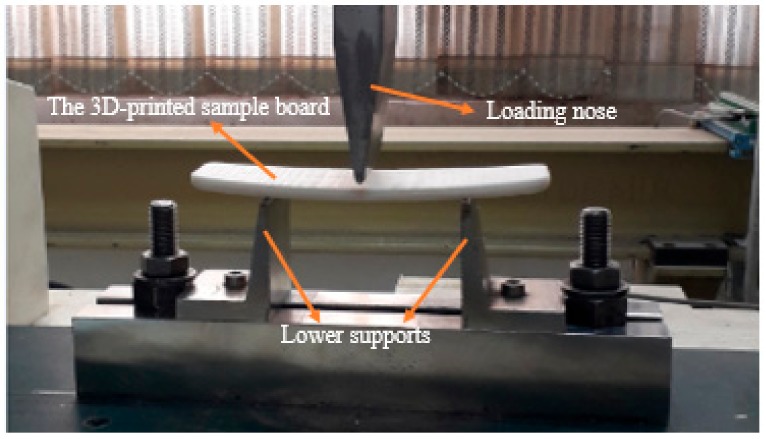
The board with a uniform honeycomb structure core under three-point bending test.

As both of these breakages are caused by bending stresses, a mechanical three-point bending test could be employed to determine the strength of the board in such loading. Stier et al. [6] also applied a similar test to their board with a novel design in shape in order to find its bending strength. The 3D-printed board with a uniform honeycomb structure in the core was tested under 3-point loading. In order to do this, the grippers of the tensile test had to be changed. The lower grip was replaced with two supports under the specimen, and the upper grip was replaced by a loading nose in the middle of the sample in order to apply force. Figure 8 shows the sample board under the three-point bending test. The test with the strain rate of 0.001 s^−^^1^ was carried out at room temperature with an 80 mm distance between two supports. A displacement-controlled test was conducted to get a maximum deflection of 4 mm in the elastic range.

### 2.5. Analytical Solution

**Figure 9:**
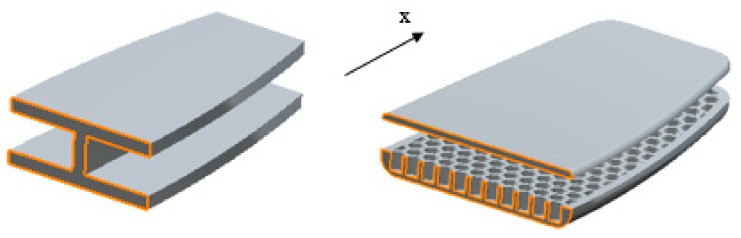
I-shaped beam and the board with equivalent sections shown with orange lines.

In this section, a geometrically linear simple approach for the analytical solution is provided to validate the experimental bending results of the 3D-printed board with a uniform honeycomb core structure. For this purpose, an equivalent I-shaped section, in which its geometrical stiffness varies along the *x*-direction, was considered to simulate the board structure, as illustrated in Figure 9.

In order to determine the deflection of the structure, strain energy methods are implemented. The density of strain energy, *u*, is expressed as:

(1) $$u=\int_{0}^{\varepsilon_{x}}\sigma_{x}\;d\varepsilon_{x}$$

The effect of bending stress is considered and formulated as:

(2) $$\sigma_{x}=\frac{M(x)y}{I(x)}$$

In this equation, *I* denotes the moment of inertia of the cross-section that varies along the *x*-direction and *M* denotes the variable moment in each section.

By substituting Equation (2) into Equation (1) and considering a linear elastic behavior, Equation (1) can be rewritten as:

(3) $$U=\int \frac{M^{2}y^{2}}{2EI^{2}}dV$$

Next, according to the second Castigliano’s method, displacements of a linear-elastic system can be determined based on the partial derivatives of the energy. Equation (4) shows Castigliano’s method [30], where $\delta_{D}$ and $F_{D}$ are the displacement and virtual or actual force at point D, respectively.

(4) $$\delta_{D}=\frac{\partial U}{\partial F_{D}}=\frac{\partial }{\partial F_{D}}\int \frac{M^{2}y^{2}}{2EI^{2}}dV=\int \frac{M}{EI^{2}}\frac{\partial M}{\partial F_{D}}y^{2}dV$$

### 2.6. Finite Element Method and Experimental Validation

In this study, a geometrically non-linear FEM software (Dassault Systemes, 6.14, Vélizy-Villacoublay, France) package ABAQUS™ was employed to numerically simulate the boards with various core structures under a three-point bending test. The computer-aided design for top shell and merged bottom shell and core were first imported into ABAQUS. For complete 3D models, especially relatively thin objects with a complex geometrical shape, mesh generation is challenging. As each board in this study not only had complex core geometry, but also had very high curvatures along the edges, tetragonal elements were implemented to successfully cover the whole geometry with good accuracy.

**Figure 10:**
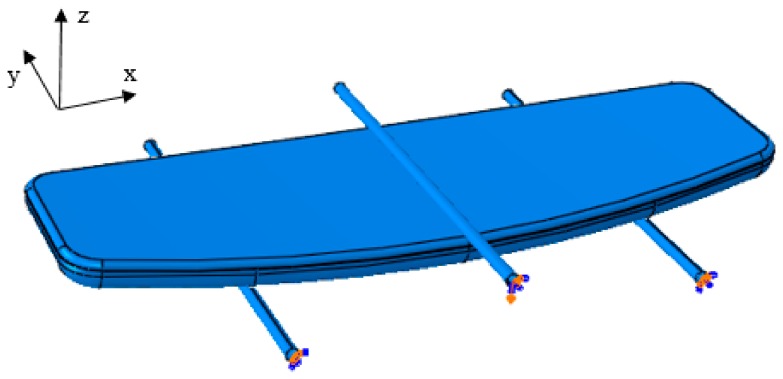
Boundary conditions of the finite element method model.

Since the top shell and bottom shell with a structural core must be meshed independently in the full solid model, it would be crucial to use the tie constraint at their interfaces to simulate impeccable bonding between both parts. The boundary conditions were chosen to be similar to the real three-point bending conditions, as numerical findings were planned to be validated experimentally. To prevent plastic deformations, boards must be maintained in the elastic regime. Therefore, in this study, all of the simulations were conducted in the elastic regime. As illustrated in Figure 10, two cylindrical supports with a radius of 2 mm, set at a 80 mm distance were completely fixed underneath the board using the encastre boundary condition, while a z-displacement of −4 mm was applied to the loading nose.

**Figure 11:**
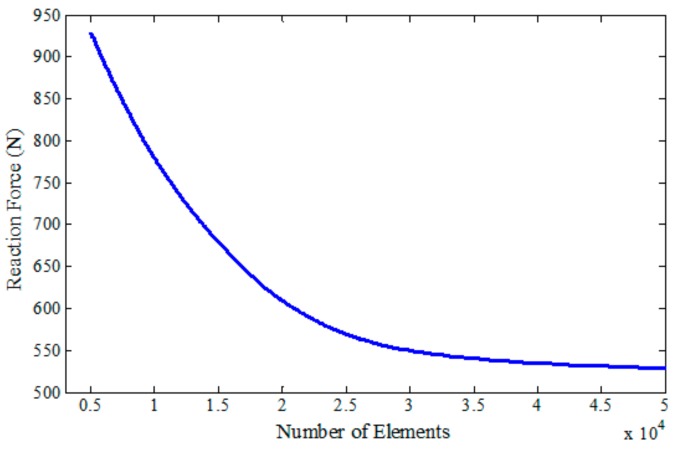
FEM mesh convergence test of the board with a uniform honeycomb core structure.

Next, in order to validate the FEM model, the designed board with a uniform honeycomb core structure under a three-point bending test was simulated. In this regard, element type C3D10M was exploited for both solid parts with approximately 37,000 elements and 2719 faces for the bottom part and 4000 elements for the top shell. To ensure the accuracy of the numerical results, a mesh sensitivity analysis was performed. In Figure 11, the reaction force of the loading nose for a maximum of 4 mm deflection was plotted versus the number of elements. It can be seen that after increasing the number of elements to >35,000, the reaction force values converge in an almost constant manner.

**Figure 12:**
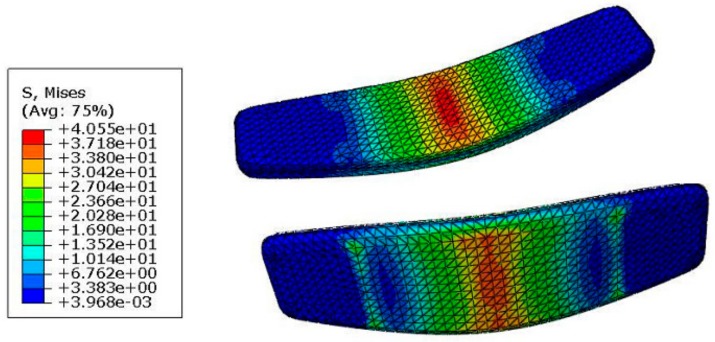
Von Mises stress contour of the board with the uniform honeycomb core.

Next, the three-point bending test was applied to the board with the uniform honeycomb core to get a maximum deflection of 4 mm. The stress contour is illustrated in Figure 12 shows the maximum stress, predictably, occurs in the middle of the board. This maximum stress is low enough (∼40 MPa) to keep the board in the desired elastic region, as the previously tested PLA material showed a yield stress level of 60 MPa.

## 3. Results and Discussions

**Figure 13:**
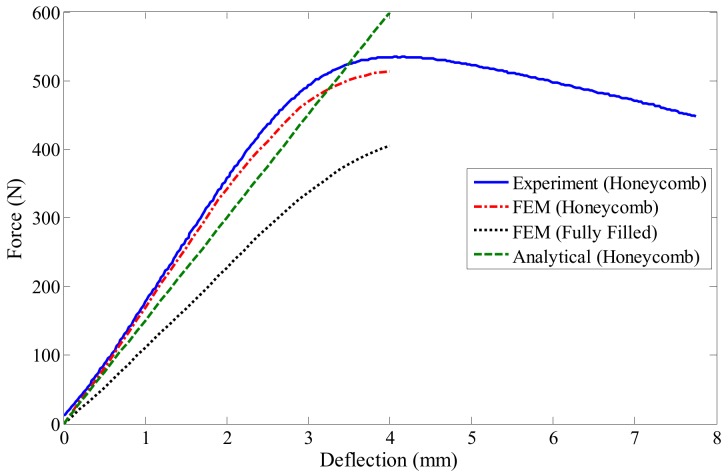
Comparison of the experimental, numerical, and analytical load–deflection curves for the three-point bending test of the honeycomb and fully-filled boards.

The force–deflection curve for the experimental, geometrically non-linear numerical, and geometrically linear analytical results are plotted and compared to each other in Figure 13. The preliminary conclusion drawn from this figure is the fact that the PLA board shows a linear elastic deformation up to 300 *N* force, beyond which the material yields, followed by plastic deformation that is manifested as a plateau after 500 *N*. From a design point of view, it is desired that the board exhibits small elastic deformations up to stress levels as large as 500 *N*. Regarding the modeling, it can be seen that, at the beginning of the deflection, the numerical and analytical results showed an excellent fit to the experimental results, but as the deflection increased, the difference between the numerical/analytical and experimental results increased. It can be seen that the geometrically non-linear FEM model can predict the non-linear experimental curve better, while the geometrically linear analytical method is unable to do so. This is particularly pronounced in the large deformation regime, revealing the importance of considering the geometrically non-linear assumption in the design of the board. However, the geometrically linear analytical method could be used as a reliable tool for predicting the behavior of the board in smaller strain ranges, circumventing an increase in computation. It is seen that at the 4 mm deflection, where the material reaches the end of the elastic region, the FEM ABAQUS predicts the experimental results very well, with an approximately 3.1% error. 

In order to statistically investigate the data dispersion between the experimental results and analytical and FEM results, the error function was defined as:

(5) $$Error=\sqrt{\frac{1}{n}\sum_{i=1}^{n}\left(Y_{i}-Y_{\exp}\right)}$$

**Figure 14:**
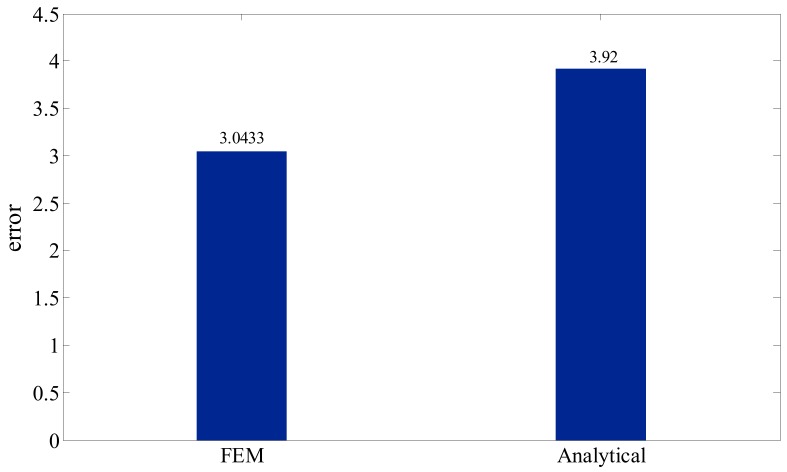
The value of the error function for the FEM and analytical results compared to the experimental results.

In this equation, ***Y*_i_** denotes the analytical or FEM results, and ***Y*_exp_** shows the experimental results. In Figure 14, the results of error function are plotted. The conclusion drawn from this plot is that the error between the experimental and FEM results was relatively low when compared to the error between experimental and analytical results; on the other hand, the finite element had less dispersion in comparison with experiment. 

### 3.1. Testing Different Core Patterns

Having validated the geometrically non-linear FEM model for the 3D-printed board with the honeycomb core structure, different designs for the core of the bottom shell were introduced to determine the structure that gives the maximal bending resistance for this particular application. All of the designed boards had the same outer frame, but different patterns were applied to the cores, while the total volume of the board and upper shell geometry was kept constant. Structures inspired by natural shapes and patterns like spiderwebs, sunflowers, pine, and carbon crystal lattices were developed. Some of them have recently attracted researchers’ attention such as triangular honeycomb (TH) [12,31] and hexagonal-rhombic (HR) [11]. For all of the structures, the mesh convergence study was conducted and the appropriate number of elements for the FEM model was selected. Furthermore, the maximum stresses of all boards with various core structures were figured to have shown a maximum stress lower than the yield stress of the PLA material.

#### 3.1.1. Hexagonal-Rhombic Structure

**Figure 15:**
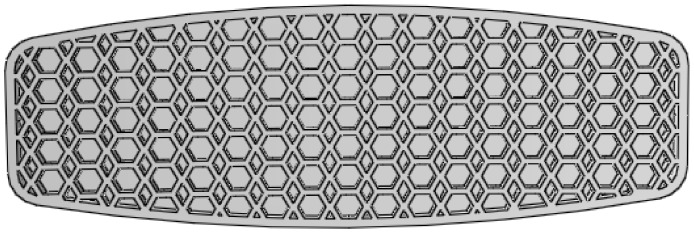
The bottom shell of the board with the hexagonal-rhombic structure.

The HR structure, which is comprised of intermeshed rows of hexagonal and rhombic patterns periodically repeated across the core of the board, has been recently presented by Platec et al. [11] as a cellular structure fabricated by an FDM technology. Having been tested under a quasi-static loading (compression) condition, this structure demonstrated a compression resistance superior to that of a uniform honeycomb. Consequently, we were motivated to investigate the bending performance of this structure for our particular application. Figure 15 presents the designed bottom shell of the board with the HR structure applied to its core.

#### 3.1.2. Triangular Honeycomb Structure

**Figure 16:**
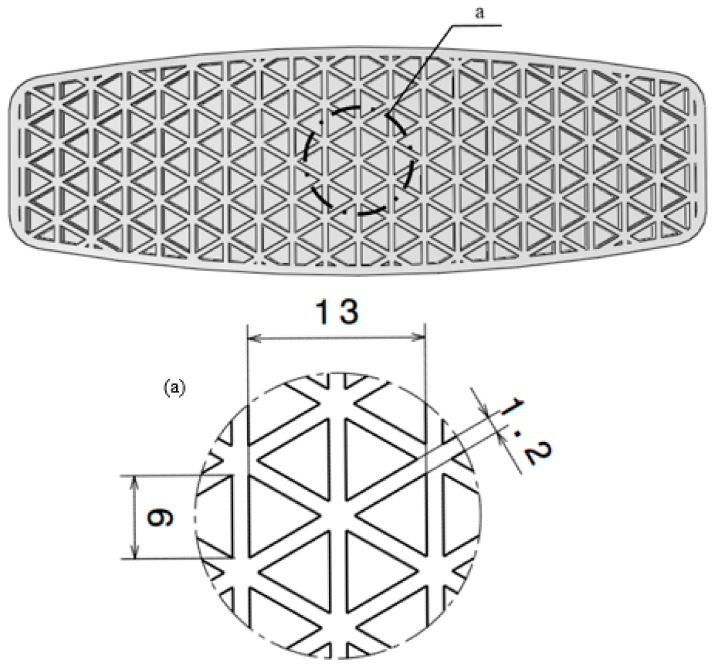
Bottom shell with the triangular honeycomb core structure and detailed view of section. (**a**) Geometry and dimensions of a TH unit-cell (all dimensions are in mm).

Recently, a composite triangular honeycomb structure was designed and manufactured by Compton et al. [12] using a 3D printing technology in order to be tested under compression. Furthermore, the ability of shape recovery of the mentioned structure was studied [27], and Bodaghi et al. [13] investigated the large deformations of TH structure by taking advantage of 3D printing technology to manufacture the samples. As the application of the TH structure is very common, we applied this pattern to the board to determine its bending resistance. The pattern is composed of repeatedly arranged hexagonal unit-cells, each comprised of equilateral triangles and shows a detailed view of geometry and dimensions of the TH unit-cell (see Figure 16).

#### 3.1.3. Hexagonal Carbon Lattice

**Figure 17:**
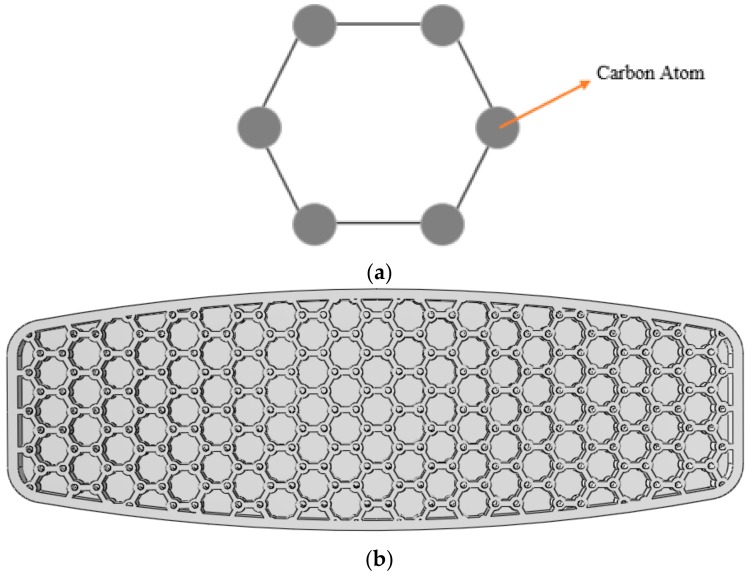
(**a**) Carbon atoms placed in the six edges of a hexagon; (**b**) Bottom shell with the carbon atom configuration structure.

There are several allotropes of carbon such as graphite, diamond, graphene, and carbon nanotubes. As illustrated in Figure 17a, in all of the noted carbon allotropes, carbon atoms (marked by black circles) are placed in vertices of regularly patterned hexagons. Inspired by this broad usage of carbon in nature, the arrangement of the carbon atoms in the edges of a hexagon was applied to the design of the core of the board. Figure 17b demonstrates the designed board where the small circles are the carbon atoms linked together by the sides of the hexagon.

#### 3.1.4. Pinecone and Sunflower-Inspired Patterns

**Figure 18:**
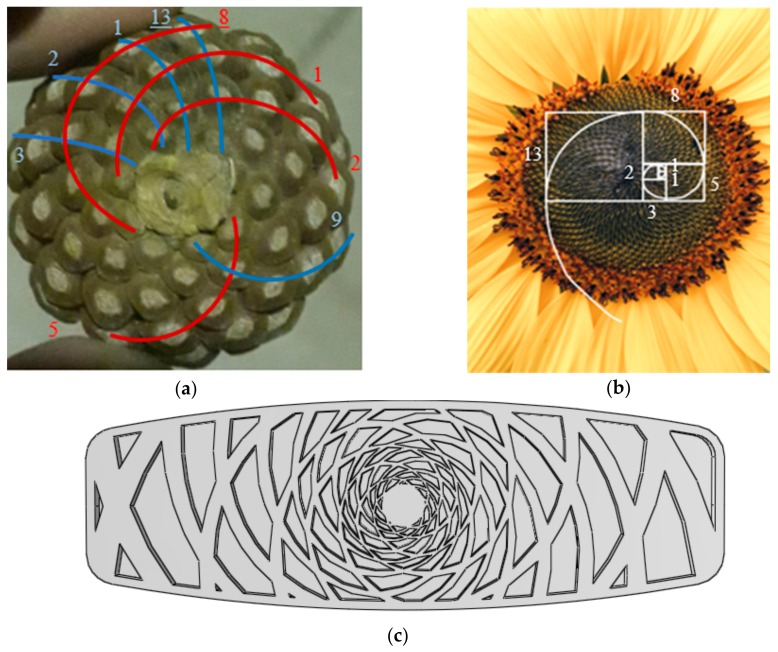
(**a**) A pinecone with two 8-number and 13-number opposite directional spirals; (**b**) Sunflower with Fibonacci spiral; (**c**) Pinecone-inspired structure designed using Fibonacci spirals.

Fibonacci numbers are a mathematical sequence starting with 1 and 1, where each number of the sequence is the summation of two previous numbers. Intriguingly, this sequence can be commonly found in nature. For instance, in a pine cone, there are a number of spirals starting from the cone center, following a spiral path to the outside of the cone. These spirals are in two opposite directions—clockwise and counter-clockwise—where the numbers of these two opposite directional spirals are the consecutive Fibonacci numbers. The 8-number clockwise and 13-number counter-clockwise spirals with red and blue colors are illustrated in Figure 18a, respectively. This phenomenon, moreover, can be found in sunflower, with Fibonacci spirals that can easily be made using squares attached to each other where the squares have consecutive Fibonacci numbers as the dimension (Figure 18b). Only by placing the seeds in the intersection of the spirals can the maximum numbers of seeds be filled in a sunflower or a pinecone. These optimizing numbers and their frequent usage in nature motivated us to design a pinecone-inspired core structure using spirals with Fibonacci numbers, as illustrated in Figure 18c.

#### 3.1.5. Spiderweb-Inspired Pattern

**Figure 19:**
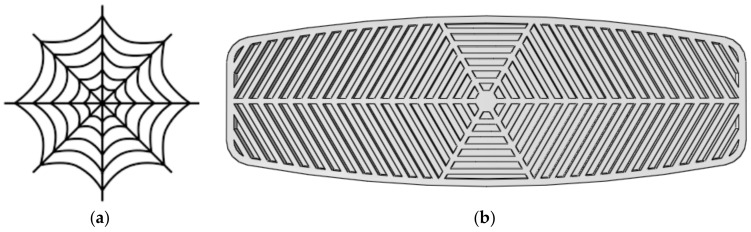
(**a**) A schematic of a spiderweb; (**b**) The spiderweb-inspired pattern applied to the core of the board.

The spider web is known to have a very high tensile strength exceeding 1 GPa. The effectiveness of the spider web should be due to the strength of the spider silk as well as the patterns of the web. As illustrated in Figure 19a, this pattern is composed of a sequence of periodic polygons that shrink as they get closer to the center of the web. The designed hexagonal pattern for the core of the board, as shown in Figure 19b, is inspired by the spiderweb.

#### 3.1.6. Functionally Graded Honeycomb Structure

**Figure 20:**
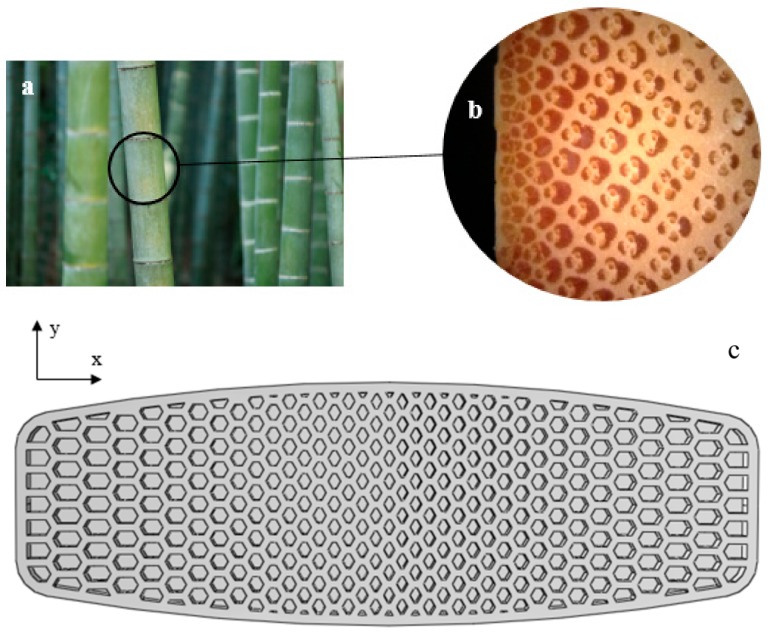
(**a**) A Bamboo stalk; (**b**) An optical image of the FG bamboo cross-section in which the dark areas are the fibers, while the light areas are the parenchyma matrices (adapted from [34]); (**c**) Graded honeycomb structure applied to the core of the board.

Bamboo, a group of perennial grasses comprised mostly of cellulose fibers and parenchyma tissue, is a natural fiber-reinforced composite that could resist harmful tropical winds (Figure 20a). Functionally graded (FG) structures are one of the most optimal choices to fabricate lightweight structures while being able to tolerate damaging stresses. In these structures, in general, the dimension of the unit-cells change with a constant gradient across the object. Near the vascular bundles of the bamboo, there are parenchyma cells, and in the cross-section illustrated in Figure 20b, the ratio of vascular bundles to parenchyma matrices decreases from the outside surface to the inside. Bamboo’s FG structure has exceptional stiffness, tensile strength, and fracture resistance [32]. These excellent properties have allowed bamboo to be used successfully in construction such as buildings and bridges, and it proved its worth when in 1991, approximately 20 bamboo houses survived the 7.5 Richter scale earthquake in Costa Rica [33].

In this section, considering the maximum stress acting in the middle of the board (see Figure 12) and being driven by excellent mechanical properties of a bio-inspired bamboo structure, we designed a FG honeycomb structure, as shown in Figure 20c, in which the dimension of the hexagonal unit-cells increases from the middle of the board across the *x*-direction (with a constant coefficient of 1.05), while the dimension of the hexagons is kept constant in the *y*-direction.

### 3.2. Results of the Different Patterns

**Figure 21:**
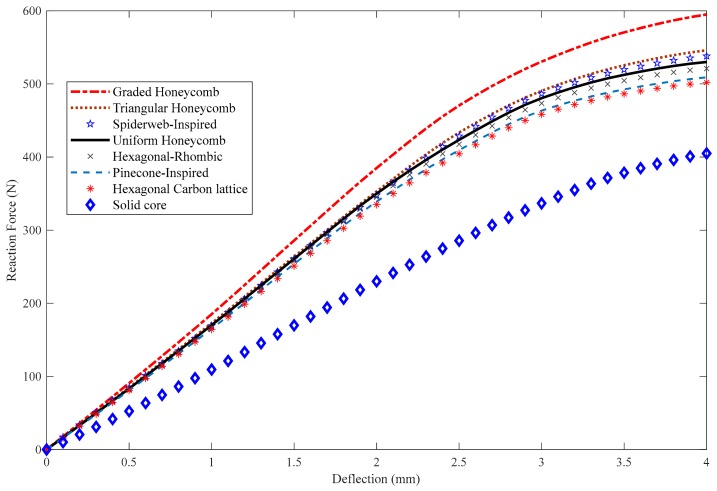
Reaction force–deflection curves for all of the tested structures.

After designing different patterns, every board with the previously mentioned core structures was applied to a three-point bending loading using the geometrically non-linear FEM software package ABAQUS. The constant displacement of 4 mm in the *z*-direction was executed by means of the loading nose, and the reaction forces for each designed board was determined. Figure 21 compares reaction force–displacement curves of different core structures.

The preliminary conclusion drawn from this figure is the fact that the FG honeycomb structure and fully filled board can tolerate maximum and minimum forces, respectively, while the rest of the patterns experienced an intermediate force. As the maximum stress occurs in the middle of the board right below the load application area, it can be found that the FG honeycomb pattern showed the best bending resistance when compared to other non-uniform patterns. For instance, the force versus deflection curve for the FG board reached a plateau after 500 N and experienced large deflections beyond this value, while the other patterned boards reached a plateau beyond 400 N. Comparing the FG and uniform honeycombs for 500 N force revealed that the functionally graded pattern could reduce the central deflection as much as 31%. It was also seen that the FG honeycomb board experienced 595 N force at 4 mm deflection and resulted in ∼12% enhancement in force compared to the uniform honeycomb structure. From the results presented in this figure, it can also be found that for a constant applied force of 400 N, the central deflection reduced by almost 97% when using the FG pattern, and was 97% lower than a board with a fully-filled core, which is a significant improvement. Furthermore, it can be concluded that the board with a uniform honeycomb pattern showed slightly better bending performance as compared to the carbon atom lattice structure, meaning that implementing circular holes did not improve the bending resistance.

## 4. Conclusions

An on-water sports board with different nature-inspired core patterns under the three-point bending test was studied in this paper. The 3D-printed sample board with a uniform honeycomb core structure was designed and fabricated from PLA using FDM 3D printing technology, and then experimentally tested under the three-point bending condition. Considering the equivalent section for the honeycomb structure, the geometrically linear analytical solution was developed for the board with a honeycomb core structure using the energy method. Furthermore, the geometrically non-linear FEM was used to simulate the sample boards under the three-point bending test. The results demonstrated coarse and fine matches between the experimental, numerical, and analytical results in the small and large deformations. Then, different structures inspired by natural patterns and shapes like spiderweb, pinecone, and carbon lattice configuration were developed and applied to the core of the board in order to determine which gave the highest bending resistance. Experimental and numerical results revealed a 31% better bending resistance of the board with the FG honeycomb pattern when compared to the board with a uniform honeycomb structure at 500 N force. Furthermore, comparing the FG with the solid core board for a fixed force of 400 N revealed, a significant, 97% reduction in the central deflection of the board.

## Figures and Tables

**Table 1:** Dimensions of the designed specimen shown in Figure 3, according to ASTM D638.

Dimensions	ASTM D638 TYPE 1
W—Narrow section width	13 mm
L—Narrow section Length	57 mm
Wo—Overall width	19 mm
Lo—Overall length	165 mm
R—Radius of fillet	76 mm
t—Thickness	5 mm
